# Exploring the Potential Link between Acute Central Serous Chorioretinopathy and Trimethylamine N-Oxide, Phoenixin, Spexin, and Alarin Molecules

**DOI:** 10.3390/biom13101459

**Published:** 2023-09-27

**Authors:** Mehmet Kaan Kaya, Sermal Arslan

**Affiliations:** Universaleye Clinic, Elazig 23040, Turkey; drsermal@hotmail.com

**Keywords:** phoenixin, spexin, alarin, chorioretinopathy

## Abstract

Purpose: Acute central serous chorioretinopathy (ACSCR) is a condition characterized by decreased visual acuity, macular thickening, and edema under the retinal layer. Although the underlying mechanisms of the disease are not fully understood, oxidative stress is considered to be a critical risk factor. The aim of this study was to shed light on the pathophysiology of ACSCR by investigating the levels of circulating trimethylamine N-oxide (TMAO), phoenixin (PNX), alarin (ALA), and spexin (SPX) molecules in ACSCR patients. Methods: The study included 30 ACSCR patients and 30 healthy individuals as controls. ACSCR was diagnosed using optical coherence tomography (OCT) imaging. Five mL blood samples were collected from all participants following overnight fasting. The levels of TMAO, PNX, ALA, and SPX in the blood samples were measured using the ELISA method. Results: Visual acuity was found to be significantly reduced in ACSCR patients compared to the control group (<0.05), while macular thickness was increased (<0.05). Furthermore, TMAO, PNX, and ALA levels were significantly higher in ACSCR patients (<0.05), while SPX levels were significantly lower compared to the control group (<0.05). In ACSCR patients, there was a positive correlation between macular thickness and TMAO, PNX, and ALA; there was, however, a negative correlation with SPX. Additionally, visual acuity was negatively correlated with TMAO, PNX, and ALA, while SPX levels decreased as visual acuity decreased. Conclusions: These results demonstrate a correlation between the TMAO, PNX, ALA, and SPX levels of ACSCR patients and their visual acuity and macular thickness. Given the role of these molecules in ACSCR’s pathophysiology, they hold promise as potential diagnostic, therapeutic, and follow-up markers in the future.

## 1. Introduction

Central serous retinopathy (CSCR) is a condition characterized by the accumulation of fluid beneath the retina, leading to severe vision loss, typically seen in working-age men [1]. It is also known as central serous chorioretinopathy (CSCR) since the source of the fluid under the retina is the choroid layer, and the disease affects the fovea, impairing central vision [2]. Active CSC is identified by the detachment of the neurosensory retina due to the accumulation of serous fluid between the photoreceptor outer segments and the RPE, often accompanied by focal changes in the RPE [3]. Patients with CSCR often experience blurred or distorted vision in one eye, a dark or dim spot in the central visual field, objects appearing smaller or farther away in the affected eye, straight lines appearing bent or distorted. Albert V Graefe first described this condition as central recurrent retinitis in 1866 [4]. CSCR is typically unilateral and affects about 80% of men between the ages of 20 and 50, with bilateral involvement in 8% of cases. This disease is most commonly observed between the ages of 30 and 50 [5,6]. Risk factors related to CSR are stress causing high dose cortisone and adrenocorticotropic hormone (ACTH) levels and hypertension, pregnancy, Cushing’s disease, etc. The exact cause of central serous retinopathy remains unclear, yet it is believed that pressure changes in the choroid layer may lead to damage of the retinal pigment epithelium (RPE), which in turn may result in the accumulation of fluid under the retina [7].

In patients with CSCR, the diagnosis is made by biomicroscopy and confirmed by fundus angiography. While the diagnosis of classic CSC can sometimes be established without fluorescein angiography, it remains a crucial component in distinguishing it from conditions, such as subretinal neovascularization, and is integral in treatment planning [8]. Optical coherence tomography (OCT) provides additional information about the presence of subretinal fluid, retinal thickening, and pigment epithelial detachments. It is also used as a non-invasive method in the follow-up of patients. The measurement of macular thickness with OCT in CSCR patients is one of the most important parameters in their clinical follow-up and was used in this study.

Current research indicates that the complex mechanism of CSCR involves the interaction of multiple factors, including genetics, type A personality, immunity, inflammatory injury, and H. pylori infection with peptic ulcer disease. Consequently, researchers have suggested and crafted specialized methods for treatment and prevention. Variability among individuals may stem from shifts in the equilibrium of gut bacteria (GM) within the human system. Some findings suggest that when the balance of GM in humans is disrupted, it may lead to the onset of conditions related to inflammation, metabolism, cognition, and immunity. Studies have reported a relationship between eye disease and GM. But the potential effect of GM on those with CSCR is still not clear. In recent studies conducted by researchers, the concept of a “gut–eye” connection was introduced and it was demonstrated that GM influences retinal conditions [9]. In another similar study conducted on diabetic rats, it was observed that the intestinal flora is associated with damage in the collected plasma and retinal samples [9].

There may be a link between the disruption of RPE and the intestinal microbiota. The intestinal microbiota, which is considered a “new metabolic organ,” plays a critical role in controlling physiological, biochemical, and metabolic processes in our bodies [9]. Dysbiosis, an imbalance in the intestinal microbiota, can disrupt the balance of many metabolic events [10]. One of the metabolites produced by the microbiota, trimethylamine N-oxide (TMAO), could be a new risk factor for CSCR [11]. Choline, L-carnitine, and betaine, found in animal foods, are converted to trimethylamine (TMA) in high amounts by anaerobic bacteria in the lumen of the gastrointestinal tract. Circulating TMA is subsequently oxidized to TMAO by the hepatic enzyme flavin monooxygenase 3 (FMO3) in the liver. TMAO produced in the intestine can also be converted back to TMA by TMAO reductase. Both TMA and TMAO can induce inflammation [11,12]. Initial observational research indicated a correlation between atherosclerosis and increased TMAO levels. Since there is a correlation between RPE and inflammation, it is worth investigating the levels of TMAO in patients with CSCR [13].

Moreover, phoenixin (PNX) is another molecule known for its anti-inflammatory properties, which was first discovered in 2013 by Yosten et al. [14,15]. Its most biologically active form consists of 14 amino acids. The precursor of phoenixin is encoded by the C4orf52 gene and synthesized on cytoplasmic ribosomes [16]. It undergoes cleavage through ectodomain shedding, a regulated process involving the cleavage of membrane proteins. PNX is highly expressed in neurons of the nucleus tractus solitarius (NTS), which are closely associated with stress circuits through connections to forebrain regions in the hypothalamic–pituitary–adrenal (HPA) axis. Additionally, PNX is found in other regions such as the paraventricular nucleus (PVN), supraoptic nucleus (SON), zona incerta, arcuate nucleus (Arc), dorsal hypothalamus, and ventromedial hypothalamus (VMH) [17,18]. It is also expressed in various organs and tissues including the heart, thymus, esophagus, stomach, duodenum, jejunum, colon, pancreatic islets, adipose tissue, ovaries, and epidermis of the skin. The subjective memory impairment group exhibited a positive correlation between the average plasma phoenixin level and immediate recall, hinting at a potential predictive role of plasma phoenixin levels in cognitive memory impairment. Recent literature indicates that phoenixin plays a role in regulating the HPG axis activity and addressing fertility issues induced by stress [14]. PNX has been shown to have protective functions in oxidative stress and inflammatory pathways, as well as playing a role in the control of food intake and thirst. Interleukin-6 (IL-6) and (IL-1β) have the potential to diminish ROS production and elevate glutathione levels in response to oxygen-glucose deprivation/reperfusion damage in BV2 microglia [14,15,17,18]. Phoenixin-14 was observed to enhance protective nitric oxide synthase and nitric oxide expression while reducing endothelial monolayer permeability in human brain endothelial bEnd.3 cells [19]. However, there is currently no research indicating whether there is a correlation between PNX levels and ACSCR.

Spexin (SPX) is a peptide molecule consisting of 14 amino acids that was first discovered in 2007 in the submucosal layer of the esophagus and stomach of organism. The prepropeptide human spexin containing 116 amino acid residues was encoded by C12ORF39 gene [20]. Subsequent studies using immunohistochemical staining methods have shown that SPX is synthesized in various biological systems and tissues such as the choroid plexus, cervical neurons, trigeminal ganglia, retinal photoreceptors, cerebellar Purkinje cells, and paraventricular and supraoptic parts of the hypothalamus [21]. It is also synthesized in other tissues, including the epidermis and adipocyte, skin, the gastrointestinal tract, liver, exocrine part of the pancreas, and adrenal cortex and medulla [22]. Spexin has recently been implicated in pain, anxiety, and depression. Another study demonstrated its involvement in regulating depression. Classical antipsychotic drugs increased spexin and POMC mRNA levels while decreasing kisspeptin-1 mRNA levels in the rat amygdala [23]. SPX plays a crucial role in weight control by suppressing food intake, restricting the intake of fatty acids, and regulating nutritional behaviors. It also contributes to stress management in biological systems [18]. A study reported there is a relationship between SPX mRNA in stress environments [21]. However, to date, there has been no study showing whether there is a relationship between SPX levels and ACSCR.

Alarin is a recently discovered 25 amino acid peptide hormone belonging to the galanin family [24,25]. Alarin mRNA is synthesized in the brain, thymus, and skin. It is also found in the aqueous and intrinsic choroidal neurons. It regulates feeding behavior, energy homeostasis, glucose metabolism, body temperature, and reproduction. Additionally, it possesses various functions such as anti-inflammatory, vasoconstrictive, and anti-edema effects for maintaining eye and skin health, along with antimicrobial activity against certain bacteria. Physiologically, alarin shows strong and dose-dependent vasoconstrictor and antiedema activity in the cutaneous microvasculature. Additionally, alarin has been shown to have effects on controlling food intake, as well as hypermetabolic and hyperthermic effects [25]. The presence of alarin activity near retinal and choroidal vessels indicates its potential role in regulating ocular blood flow, as it might influence the blood vessel caliber. In patients with diabetic retinopathy, there is an increase in the amount of plasma and aqueous alarin [26].

In CSCR, fluid accumulation under the retinal layer may cause damage to the retinal pigment epithelial (RPE) cells, leading to retinal degeneration and eventually blindness [7]. This study aimed to investigate, for the first time, the relationship between trimethylamine N-oxide (TMAO), a molecule associated with inflammation, and three peptides: phoenixin (PNX), which has anti-inflammatory properties, spexin (SPX), which contributes to stress management, and alarin, which has anti-edema activity, in patients with acute CSCR (see Table 1).

## 2. Materials and Methods

**Figure 1:**
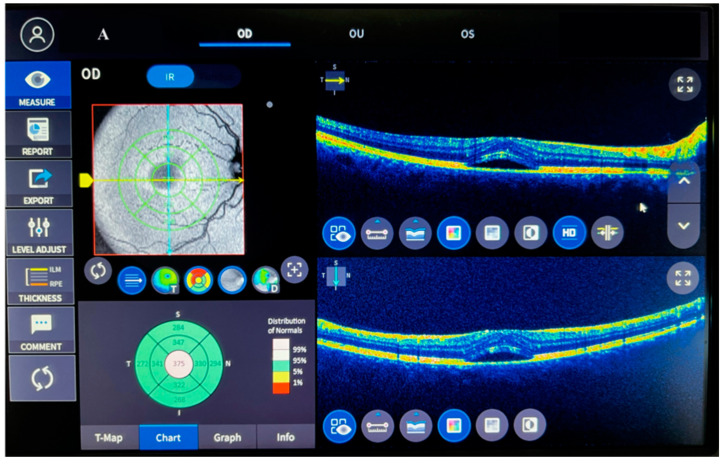

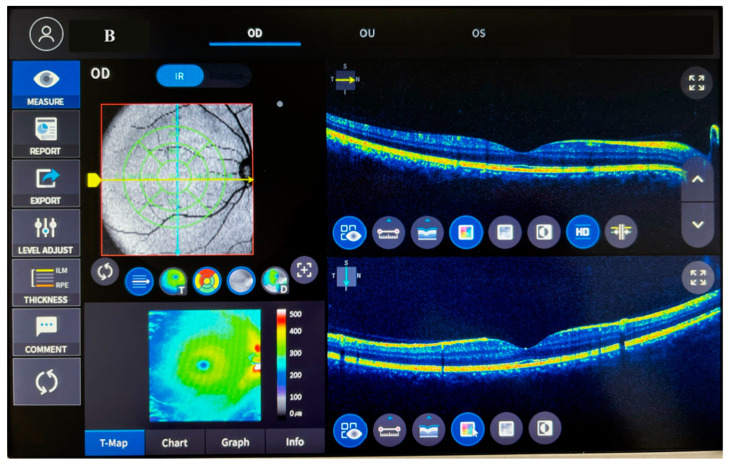
Comparison of left eye macula between controls (**A**) and acute central serous chorioretinopathy patients (**B**).

This study was conducted in collaboration with a faculty member from the Universal Eye Hospital and the Department of Medical Biochemistry at Fırat University Faculty of Medicine, with the approval of the non-interventional research ethics committee of Fırat University dated 26 January 2023 (session number 2023/02-28). The participants were provided with information about the study, and their consent was obtained before enrollment. The study adhered to the ethical standards outlined in the 1983 revision of the Declaration of Helsinki. The study included 30 participants diagnosed with acute central serous chorioretinopathy (ACSCR) by an ophthalmologist, as well as 30 healthy control participants who were recruited from volunteers without any medical issues and identified during their annual check-ups. ACSCR diagnosis was established using detailed ophthalmological examination, fundus fluorescein angiography (FFA) evaluation, and optical coherence tomography (OCT) (see Figure 1).

The study involved taking medical history and performing physical examinations on all participants. Patients with certain pre-existing conditions such as COPD, liver disease, acute MI, diabetes mellitus, corticosteroid use, sleep apnea, stressful life events, *Helicobacter pylori* infection, hypertension, renal failure, thyroid issues, cardiac cachexia, morbid obesity, those below 18 or above 80 years of age, those with active infections, and those with a history of cerebrovascular disease were excluded from the study. The participants’ BMI was calculated by dividing their weight in kilograms by their height in meters. Blood samples of 5 mL were collected from all participants according to specified guidelines, and those samples were centrifuged at 4000 rpm for 5 min. The samples were divided into 5 equal parts and stored in Eppendorf tubes at −40 °C until analysis.

### 2.1. Measurement of Molecules with ELISA

The studied molecules, including PNX (SunRed, Biological Technology Co., Catalog no: 201-12-927) Shanghai, China) [27], TMAO (SunRed, Biological Technology Co., Catalog no; 201-12-7378 Shanghai, China) [28], SPX (catalog # E3507Hu, Bioassay Technology Laboratory, Shanghai, China) [27], and ALA (galanin-Like peptide) (Sunred Bioscience, Catalog no: 201-12-5592 Shanghai, China) [26], were measured using ELISA kits. These commercial kits were used to quantify the molecules in accordance with their respective procedures. Plate washings were performed using an automatic washer, Bio-Tek ELX50 device (BioTek Instruments, Winooski, VT, USA), while absorbance readings were obtained using a ChroMate Microplate Reader P4300 ELISA reader (Awareness Technology Instruments, Palm City, FL, USA) at a wavelength of 450 nm. The PNX kit had a minimum detection limit of 0.001 ng/mL, the TMAO kit had a minimum detection limit of 0.043 ng/mL, the SPX kit had a minimum detection limit of 0.10 pg/mL, and the ALA kit had a minimum detection limit of 0.214 ng/mL. All kits had intra-assay coefficient variation (CV) values less than 10%, and inter-assay (variation between days) CV values less than 12%.

### 2.2. Statistical Analysis

Statistical analyses were conducted using the computer software package SSPS version 22. Descriptive statistical methods such as the mean and standard deviation were used to evaluate the study data. For comparisons of quantitative data with normal distribution, parametric tests such as Student’s t-test were used. One-way analysis of variance (one-way ANOVA) was used for comparisons between groups. The Wilcoxon paired two-sample test was used to test the significance of differences between two paired samples. The results were evaluated at a 95% confidence interval, with a significance level of *p* < 0.05.

## 3. Results

**Figure 2:**
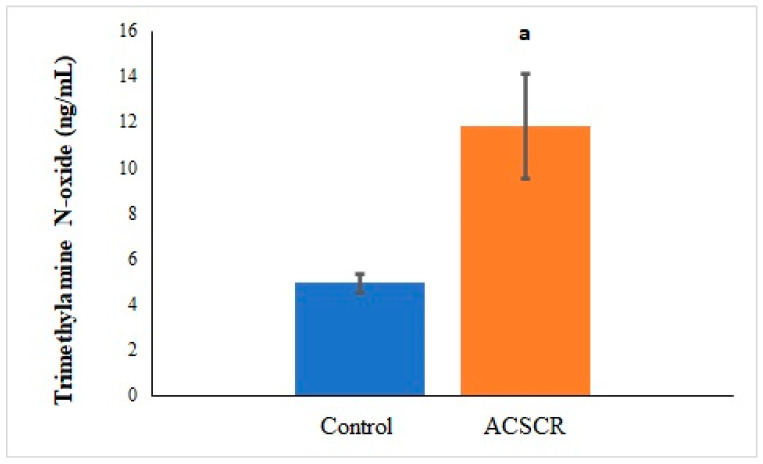
Comparison of trimethylamine N-oxide values of patients with acute central serous chorioretinopathy and controls. ACSCR: acute central serous chorioretinopathy. TMAO: trimethylamine N-oxide. a: control group versus ACSR group (*p* < 0.05).

**Figure 3:**
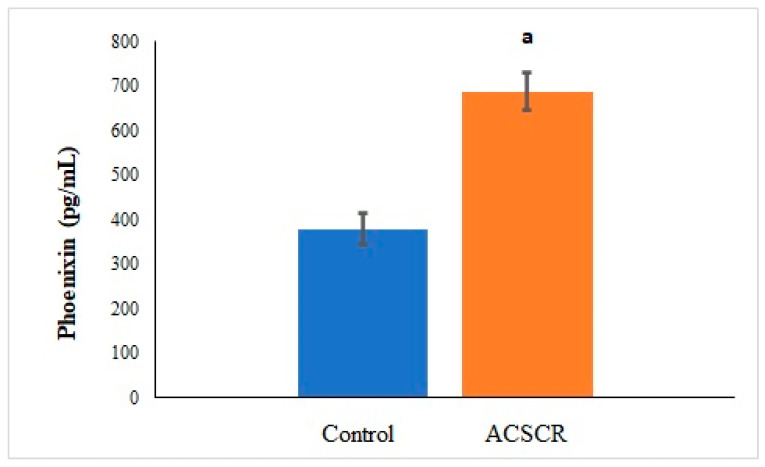
Comparison of phoenixin values of patients with acute central serous chorioretinopathy and controls. ACSCR: acute central serous chorioretinopathy. PNX: phoenixin. a: control group versus ACSR group (*p* < 0.05).

**Figure 4:**
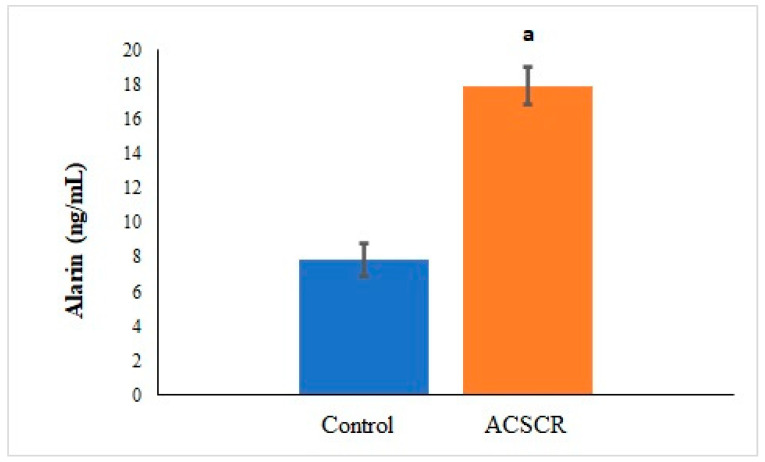
Comparison of alarin values of patients with acute central serous chorioretinopathy and controls. ACSCR: acute central serous chorioretinopathy. ALA: alarin. a: control group versus ACSR group (*p* < 0.05).

**Figure 5:**
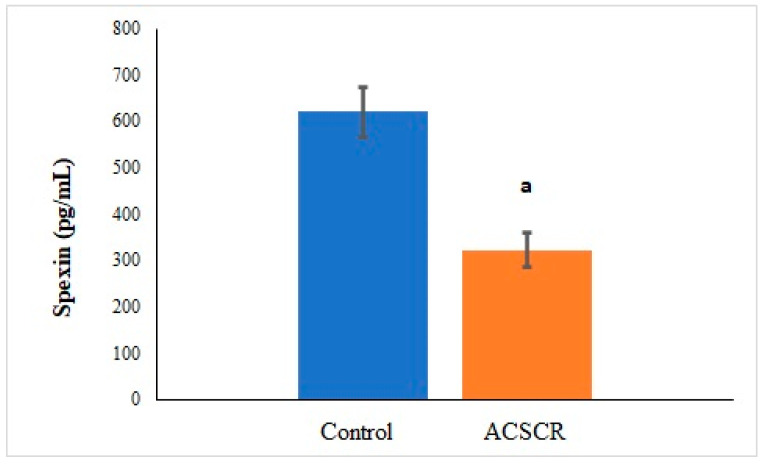
Comparison of spexin values of patients with acute central serous chorioretinopathy and controls. ACSCR: acute central serous chorioretinopathy. SPX: spexin. a: control group versus ACSR group (*p* < 0.05).

The levels of TMAO (*p* < 0.05), PNX (*p* < 0.05), and ALA (*p* < 0.05) were significantly higher in ACSCR patients compared to the control group, while the levels of SPX were significantly lower (*p* < 0.05) (Figure 2, Figure 3, Figure 4 and Figure 5). These increases and decreases were about twice as different. In other words, the increases in TMAO (Figure 2), PNX (Figure 3), and ALA (Figure 4) were about two times higher in ACSCR patients compared to the control group, while the decrease in SPX was also about two times higher (Figure 5). Positive correlations were found between macular thickness and TMAO, PNX, and ALA in ACSCR patients, while a negative correlation was found between macular thickness and SPX. Additionally, a negative correlation was found between visual acuity and TMAO, PNX, and ALA in ACSCR patients, while a positive correlation was noted between visual acuity and SPX. Further details about these correlations are provided in Table 2.

## 4. Discussion

This study found that TMAO levels were significantly higher in patients with ACSCR compared to the control group. The exact cause of choroidal hyperpermeability and fluid leakage in CSCR is still unknown; however, it may be related to factors such as mineralocorticoid receptor activation, venous congestion, and inflammation. Recent studies suggest that inflammation triggers the development of CSCR. TMAO, which is produced by the gut microbiota, has been associated with various metabolic and inflammatory disorders [29]. Therefore, the significant increase in TMAO levels in ACSCR patients in this study may be a trigger for inflammation and the development of ACSCR disease [30]. A study by Matet et al. reported that serum lipocalin 2 (an anti-inflammatory acute-phase reactant) levels were decreased in patients with acute and chronic CSCR compared to the control group [31]. Furthermore, this study found that the systolic blood pressure of ACSCR patients was higher than the control group, although it was not statistically significant. It has been reported that hypertension is associated with a higher risk of developing CSCR (odds ratio: 2.25–2.3) [32]. Some forms of hypertension have also been linked to the gut microbiota, and TMAO has been shown to cause vasoconstriction and increase systolic blood pressure [33,34]. Therefore, the increased TMAO levels in ACSCR patients in this study may also be associated with their partially higher systolic blood pressure readings.

Moreover, this study revealed that ACSCR patients had significantly increased blood PNX values compared to the control group. This increase in PNX values is likely due to the compensatory response of the retinal pigment epithelium to counteract the stress and inflammation caused by the accumulation of fluid under the retina. Previous studies have shown that PNX can reduce physiological stress, similar to cortisol, and can also reduce lipopolysaccharide-induced inflammation [35,36]. In other words, PNX can control inflammation by decreasing levels of TNF-α and interleukins [37]. Additionally, PNX has protective effects on oxidative stress and inflammatory pathways induced by psychological and physiological stress [14,38]. Since ACSCR is associated with an oxidative stress-related risk factor, PNX, a new stress indicator, may play a role in the pathophysiology of ACSCR disease [39].

This study found that, like TMAO and PNX, ACSCR patients had significantly increased levels of blood ALA. In ACSCR, the accumulation of fluid under the retina results in oxidative stress due to the deterioration of the retinal pigment epithelium. The rise in blood alarin levels, as reported in this study, may be a response to eliminate this oxidative stress. Studies suggest that ALA can prevent fibrosis by decreasing oxidative stress and can also reduce energy dysregulation in tissues [40]. Alarin is involved in energy homeostasis. Therefore, ALA may contribute to maintaining the energy balance necessary for vision [41]. Furthermore, increased ALA levels in ACSCR may decrease edema, as a study reported that ALA exhibited strong and dose-dependent vasoconstrictor and anti-edema properties in cutaneous microvasculature [24]. ALA may also contribute to the partial increase in systolic pressure in ACSCR, as it has a vasoconstrictive effect.

This study found that ACSCR patients have significantly increased levels of circulating TMAO, PNX, and ALA, while the amounts of SPX decreased significantly. SPX is a molecule that regulates nutrition and metabolism by affecting hypothalamic neurons centrally and peripherally to maintain energy homeostasis [42]. In ACSCR, the accumulation of fluid under the retina due to the deterioration of the retinal pigment epithelium causes oxidative stress and metabolic changes. These changes can disrupt energy balance and the fluid inflow–outflow equilibrium, leading to macular edema [43]. A study showed that glucose could stimulate SPX secretion from a pig pancreas [44]. The reduction in circulating SPX levels observed in this study may be a result of strict control of carbohydrate metabolism to restore energy homeostasis (glucose requirement) and water and osmotic regulation in the retina to alleviate edema [45]. Furthermore, a study showed that SPX treatment could improve insulin resistance, dyslipidemia, oxidative stress, inflammation, apoptosis, and fibrosis [46]. Given that ACSCR is associated with oxidative stress and inflammation, the reduced levels of circulating SPX in this study may be due to the endogenous production of SPX to counteract these harmful effects [47]. However, as there are no other studies investigating this phenomenon, we cannot make any comparisons with other data. Thus, the underlying mechanism behind the reduction in SPX levels in ACSCR patients remains an important area for further research.

## 5. Conclusions

TMAO, PNX, ALA, and SPX changes are thought to potentially play a role in the pathophysiology of ACSCR. Therefore, in the future, reducing the levels of TMAO, PNX, and ALA and administering SPX may be potential therapeutic strategies for controlling this disease. Simultaneously evaluating mRNA expression levels of the PNX, ALA, and SPX genes holds promising potential for future scientific investigations. 

## Figures and Tables

**Table 1:** Comparison of some demographic characteristics, central macular thickness, visual acuity, and glucose.

Parameters	Control	ACSCR	*p*
Age	47.4 ± 8.22	49.32 ± 5.16	0.81
Gender (male/female)	22/8	23/7	0.94
BMI	24.7 ± 1.6	24.9 ± 2.3	0.87
Glucose (mg/dL)	94.8 ± 5.33	99.6 ± 5.22	0.77
CMT (μm)	216.92 ± 12.34	476.11 ± 72.41	0.001
VA	1.00 ± 0.00	0.52 ± 0.31	0.026

ACSCR: acute central serous chorioretinopathy. CMT: central macula thickness. VA: visual acuity (snellen).

**Table 2:** Correlations between studied parameters of study groups.

Correlations	r Value	*p* Value
TMAO-PNX	0.494	0.011
TMAO-SPX	−0.582	0
TMAO-ALA	0.528	0
PNX-ALA	0.622	0
PNX-SPX	−0.764	0
SPX-ALA	−0.587	0
TMAO-CMT	0.612	0
PNX-CMT	0.498	0.011
ALA-CMT	0.576	0.001
SPX-CMT	−0.542	0.001
TMAO-VA	0.498	0.011
PNX- VA	0.578	0.001
ALA-VA	0.612	0
SPX-VA	0.562	0
CMT-VA	−0.596	0

## Data Availability

The data presented in this study are available in this article.
